# Antioxidant Therapy in Neonatal Hypoxic Ischemic Encephalopathy: Adjuvant or Future Alternative to Therapeutic Hypothermia?

**DOI:** 10.3390/metabo14110630

**Published:** 2024-11-16

**Authors:** Veronica Notarbartolo, Bintu Ayla Badiane, Vita Maria Angileri, Ettore Piro, Mario Giuffrè

**Affiliations:** 1 Neonatology and Neonatal Intensive Care Unit, University Hospital “Paolo Giaccone”, 90127 Palermo, Italy; 2 Department of Health Promotion, Mother and Child Care, Internal Medicine and Medical Specialties, University of Palermo, 90127 Palermo, Italy; bintuayla.badiane@community.unipa.it (B.A.B.); ettore.piro@unipa.it (E.P.); mario.giuffre@unipa.it (M.G.); 3 Neonatal Intensive Care Unit with Neonatology, “G.F. Ingrassia” Hospital Unit, 90131 Palermo, Italy; vita.angileri@asppalermo.org

**Keywords:** therapeutic hypothermia, asphyxia, hypoxic ischemic encephalopathy, antioxidants, oxygen reactive species (ROS), hypoxia, pro-oxidant

## Abstract

Background: Oxidative stress-related diseases in newborns arise from pro-oxidant/antioxidant imbalance in both term and preterm neonates. Pro-oxidant/antioxidant imbalance has shown to be present in different pathological conditions such as hypoxic ischemic encephalopathy (HIE), retinopathy of prematurity (ROP), bronchopulmonary dysplasia (BPD), necrotizing enterocolitis (NEC), and patent ductus arteriosus (PDA). Methods and Results: We performed a narrative review according to the most recent available literature (2012–2024), using Scopus and PubMed as electronic databases. Many observational and experimental studies in vitro and in vivo have evaluated the effectiveness of antioxidant therapies such as melatonin, erythropoietin (EPO), allopurinol, N-acetylcisteine (NAS), and nitric oxide synthase (NOS) inhibitors in these diseases. Perinatal asphyxia is one of the most important causes of mortality and morbidity in term and near-term newborns. Therapeutic hypothermia (TH) is the gold standard treatment for neonates with moderate-severe perinatal asphyxia, resulting in a reduction in the mortality and neurodevelopmental disability rates. Conclusions: According to the most recent literature and clinical trials, melatonin, allopurinol, NAS, NOS inhibitors, magnesium sulfate, and stem cells stand out as promising as both adjuvants and future probable alternatives to TH in the treatment of HIE.

## 1. Introduction

Oxidative stress reflects an alteration in the balance between free radical production and their neutralization. The antioxidant defense system includes both enzymatic (i.e., reduced glutathione, GSH; superoxide dismutases, SODs) and non-enzymatic mechanisms (i.e., ceruloplasmin; vitamins), playing a fundamental role in reducing cellular damage [1]. Several health problems in newborns are a consequence of an altered balance between free radical production and antioxidant defense mechanisms [2]; the result is an oxidative cellular damage that causes an entity known as “oxygen radical disease of neonatology” [2,3], whose clinical manifestations differ according to which organ is mostly affected (retinopathy of prematurity, ROP; bronchopulmonary dysplasia, BPD; necrotizing enterocolitis, NEC; patent ductus arteriosus, PDA; hypoxic ischemic encephalopathy, HIE) [4]. Previous studies have, in fact, already demonstrated that inflammation and oxidative stress are at the basis of newborns’ common morbidities development [5,6,7]. During an asphyxiation event, reactive oxygen species (ROS) and reactive nitrogen species (RNS) are produced, creating an imbalance between oxidizing enzymes and antioxidants [8,9]. Premature birth is a risk factor for oxidative stress because antioxidant defense mechanisms develop in the last trimester of pregnancy. The improvement of antioxidant defense systems in term neonates and the lower incidence of pathological conditions that increase oxidative stress in this category of newborns means that preterms are at greater risk of having lower levels of antioxidant defense markers [10]. Moreover, Boskabadi et al. [11], in their prospective study, highlighted how the serum pro-oxidant/antioxidant (PAB) level was significantly lower in preterm neonates rather than in term ones (*p* < 0.001). In the uterus, the partial pressure of oxygen is extremely low (25–30 mmHg); after birth, it rapidly increases up to 75–85 mmHg. Therefore, the fetal-neonatal transition involves the appearance of inevitable oxidative stress that the organism must try to limit by using the antioxidant defense systems it has developed [12,13,14,15].

During the perinatal period, when cerebral flow is acutely or sub-acutely interrupted, the result is multi-organ failure [14]: animal experimental models have demonstrated how perinatal asphyxia and the use of oxygen in the reanimation process can produce brain injury [15]. HIE incidence is about 1.5–2 per 1000 live births [12] and is higher in low-income countries [8], up to 26 per 1000 live births [16]. A total of 0.75 million babies suffers from a moderate to severe form of HIE worldwide, and the result is that 400,000 babies have a consequential neurodevelopmental impairment [12]. HIE can affect both preterm and term neonates with different characteristics: periventricular leukomalacia and germinal matrix bleed in the first group, and watershed-predominant pattern in the second [17]. Today, HIE remains the major cause of death in term neonates worldwide [8]. Both antepartum (i.e., maternal fever, prolonged rupture of membranes) and intrapartum risk factors (i.e., uterine rupture, shoulder dystocia) can be considered as “sentinel events” for HIE development [8]. Therapeutic hypothermia (TH) is the gold standard for moderate and severe forms of HIE in late-preterms and term neonates [13], but the aim of this review was to evaluate experimental therapies that could be used along with the standard of care for HIE in this category of newborns.

## 2. Materials and Methods

We performed a narrative review according to the most recent available literature (2012–2024). English clinical trials, original papers, reviews, and meta-analyses were included. We excluded case reports, letters, and series. The following keywords (alone or in combination) were used: oxidative stress, newborns, children, preterms, term, late-preterm, perinatal asphyxia, hypothermia, hypoxic ischemic encephalopathy, oxygen, neonatal intensive care unit, melatonin, allopurinol, N-acetyl-L-cysteine, resveratrol, lactoferrin, EPO, NOS inhibitors, docosahexanoic acid, edaravone, magnesium sulfate, stem cells, sovateltide, vitamins C and E, lutein. The electronic databases used were PubMed and Scopus.

## 3. Neonatal Hypoxic Ischemic Encephalopathy

HIE is defined as a condition where cerebral blood flow is interrupted with a consequent lack of oxygen to the affected area. The result is a switch of metabolism from aerobic to anaerobic, with the consequent depletion of high energy metabolites (“primary energy failure”) [18]. Effective prevention and treatment are essential not only for reducing global financial burdens, but more importantly, for safeguarding neonatal health [19]. Therefore, it is important to understand the underlying pathophysiological mechanisms and to intervene in the latency period of approximately 6 h before a “secondary energy failure” is triggered. Secondary energy failure is characterized by a second high-energy metabolite decrease; additional features of this phase are oxidative stress, the accumulation of toxic metabolites, and delayed apoptosis [18]. The oxidant products that are generated after resuscitation include free radicals (i.e., nitric or carbonic species) that can react with other radicals, forming molecules that can decompose into toxic products [9]. The presence of perinatal sentinel events is generally associated with an unfavorable outcome and a greater need for resuscitation at birth [20].

### 3.1. Definition

HIE can develop during the prenatal, perinatal, or postnatal period; its effect on brain injury is different depending on whether it affects preterm or full-term newborns. In particular, in term neonates, the basal ganglia and thalamus are mainly involved along with the more peripheral brain areas (“watershed-pattern”). In contrast, in preterms, HIE mainly results in white and grey matter injury in different brain areas [21]. After a hypoxic-ischemic event, a partial healing process takes place within 1 h; then, in the next 6 h, oxidative metabolism and inflammation arise (see below). Six to forty-eight hours after an asphyxiation event, the release of excitatory neurotransmitters and free radicals occurs; finally, there is a remodeling process in the injured brain [22]. Before apoptotic brain damage occurs, there is a time-window of the first 6 h, in which we have to act from a therapeutic point of view, to reduce the morbidity and mortality rates associated with HIE in newborns [8].

### 3.2. Pathogenesis of HIE

**Figure 1:**
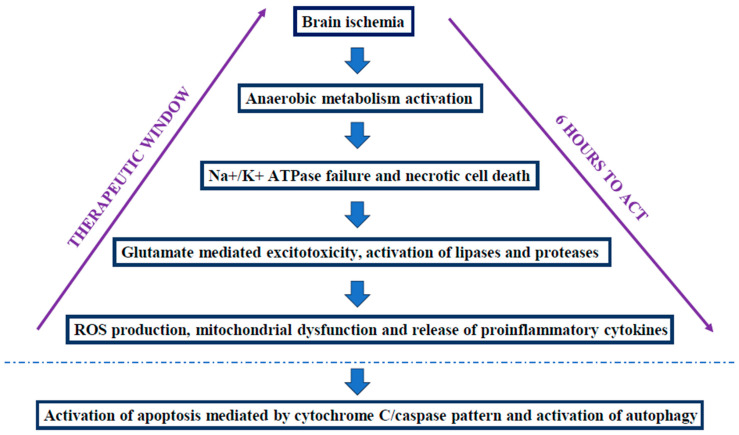
Pathophysiological steps of HIE. ROS: reactive oxygen species.

During a brain ischemia, several biochemical events take place as a result of a shift toward an anaerobic metabolism, with the consequent glycolysis activation that contributes to a rapid depletion of cerebral glucose [8]. A loss of adenosine triphosphate (ATP) and ATP-dependent ion transport pumps occurs, with cells undergoing necrosis. The result is an upregulation of the excitotoxicity neurotransmitter receptors (i.e., glutamate, α-amino-3-hydroxyl-5-methyl-4-isoxazole-propionate, AMPA, and N-methyl-D-aspartate, NMDA) [8,23]. During a hypoxic-ischemic insult, the basal ganglia and thalamus are more predisposed to damage in term neonates because they are abundant in glutamate receptors [8,23]. The increase in glutamate levels triggers proteases and lipase activation, with a consequent release of arachidonic acid, free fatty acids, superoxide free radicals, and prostaglandins [24]. Oxidative stress is more frequent in the neonatal brain due to greater vulnerability to free radicals: this is the consequence of the richness in the fatty acids and iron in the neonatal brain. These elements have a pivotal role in ROS formation [23]. ROS production causes an important change in the mitochondria, with ischemic starvation, dysfunction, and delayed neuronal death [19,23]. Reperfusion injury is due to a contemporary compensatory increase in cerebral blood flow mediated from nitric oxide synthase (NOS) [8]. This last one, along with dihydronicotinamide-adenine dinucleotide phosphate (NADPH) oxidases (NOX), are the main systems of ROS production; in particular, the role of NOX is controversial because they seem to worsen the neurological outcomes in animal models [23]. Oxidative stress creates a “susceptibility window” for HIE [23]: after the activation of the resident immune cells in the brain (i.e., microglia), with an increase in nitric oxide (NO), glutamate, and ROS levels, a strong production of inflammatory cytokines (i.e., interleukin-1α, IL-1α, IL-6, IL-8) takes place, resulting in neuronal apoptosis, decreased microvascular flow, and the worsening of brain injury [23]. The greater the cytokine production, the greater the HIE severity [19]. When the energy supplies decrease significantly, the apoptotic process takes place with cytochrome-C/caspase-3 cascade activation [25]; apoptosis can precede mitochondrial dysfunction, with nuclear factor kappa-light-chain-enhancer of activated B cells (NF-kB) pathway activation. When energy supplies are exhausted, necrotic cell death occurs [21]. Finally, another mechanism that is HIE-mediated is autophagy, a cellular protection mechanism that helps to restore homeostasis when oxidative stress arises, but when dysregulated, it can contribute to cellular damage [19]. Figure 1 shows a schematization of the pathophysiological steps of HIE.

### 3.3. Potential Biomarkers of HIE

Some free radical biomarkers can be used to identify neonates at higher risk of moderate-severe forms of HIE: an increase in cord blood SOD and catalase (CAT) concentrations, which are the first-line antioxidant defenses, is positively associated with the Sarnat score, but reference values are still not available. The same considerations can be made for the NO and malondialdehyde (MDA) serum levels, which are important lipid peroxidation markers [8]. Moreover, non-protein-bound iron (NPBI), whose plasmatic concentration is associated with severe forms of HIE, can be used as a marker of ROS production [26]. Normal cord values are <6.91 μmol/L. An increase in the urinary excretion of uric acid (uUA) is associated with oxidative brain damage: uUA/uCR (urinary excretion of creatinine) ratio > 2.3 mg/mg in the first 72 h is strictly diagnostic of severe HIE [27]. Finally, the levels of advanced oxidation protein products (AOPPs) are also correlated with severe forms of HIE (normal cord values < 80.39 μmol/dL) [26,28]. El-Mazary et al. [29] showed that a reduction in the serum selenium levels was associated with HIE. Moreover, a recent longitudinal retrospective study conducted by Lee et al. [13] demonstrated that unconjugated bilirubin (UB) levels were correlated with clinical stage, imaging findings, and the neurodevelopmental outcomes of newborns affected by HIE at birth. In particular, in the mild form of HIE, the bilirubin levels were higher before hypothermia treatment, perhaps due to its potential neuroprotective role against selective necrosis in specific brain tissues (i.e., basal ganglia, thalamus). The consequence is that bilirubin can be used as a free radical scavenger during neonatal HIE, as demonstrated by Haga et al. [30]. Finally, another important brain injury marker is the S100B protein: this cytosolic calcium-binding protein plays a fundamental role in maintaining cellular homeostasis in the central nervous system. An increase in its serum levels concentrations in the first 24 h of life can predict brain damage in asphyxiated newborns [31].

### 3.4. Therapeutic Hypothermia (TH)

Whole-body TH is part of the standard of care for *late-preterms* and term newborns affected by moderate and severe forms of HIE; it must be started within 6 h of the event [32]. The positive effects of TH are also maintained in early childhood, with a higher percentage of children surviving without neurological outcome and an intelligence quotient (IQ) > 85 compared to the untreated control group (*p* = 0.04) [20,33]. The latest recommendations of the Italian Society of Neonatology [34] on the treatment of HIE suggest treating (strong criterion) all newborns > 35 wks, >1800 gr and within 6 h of life, in the presence of asphyxia (criterion A), neurological examination abnormalities (criterion B), and amplitude integrated electroencephalography (aEEG)/EEG anomalies (criterion C). These recommendations are the result of a review of the care practices currently in use in this area [35,36,37,38].

Criterion A:

- An Apgar score ≤5 at 10 min of life; or
- Need to continue neonatal resuscitation using invasive measures and not yet at 10 min of life; or
- Fetal or neonatal acidosis on cord blood gas analysis or on any blood gas analysis obtained in the first 60 min of life (pH ≤ 7; BE ≥ −12 mmol/L).

Criterion B:

At least two of the following signs:

- Lethargy/coma;
- Reduced/absent motility;
- Altered posture;
- Hypotonia/flaccidity;
- Incomplete/weak/absent primitive reflexes;
- Pupillary anomalies.
- Seizures

Criterion C:

- Electrical activity moderately abnormal (upper edge > 10 microV and lower edge < 5 microV) or severely abnormal (upper edge < 10 microV and lower edge < 5 microV);
- Electrical crises;

or

- Discontinuous or inactive electroencephalographic tracing [34].

Even in the presence of only criteria A and B, treatment is recommended [34]. For all other hypotheses, see Table 1 ([34,35] modified). The cooling phase (rectal temperature 33.5–34 °C) lasts 72 h, then a slow and gradual warming begins (0.3–0.4 °C/hour) in order to avoid the onset of convulsions until the rectal temperature reaches 36.5–37 °C [21].

## 4. Antioxidant Therapies in Neonatal HIE

### 4.1. Melatonin

Melatonin (N-acetyl-5-methoxytryptamine) is an indolaminic hormone that is mainly produced by the pineal gland [39,40]. Melatonin has several key functions such as regulating circadian patterns (thermoregulation and sleep–wake cycles), seasonal reproduction, glucose levels, and increasing the immune system. It has hormonal, paracrine, autocrine, antioxidant, and free radical scavenger roles [41]. Melatonin has favorable pharmacokinetics and is safe and widely available. Pineal melatonin production is not fully developed in newborns and is still immature even in term infants for 2–4 months. This immature production causes a transitory melatonin shortage at birth, which makes the infant more vulnerable to oxidative injury [42]. Receptor and non-receptor mechanisms mediate the above-mentioned melatonin neuroprotection activities [39,42]. Through its ability to cross the blood–brain barrier and reach the intracellular compartment, it can help the process of preserving nerve cells to prevent or retard the progression of disease in the nervous system in hypoxic ischemia. Furthermore, it decreases microglial and astrocyte proliferation and supports subsequent myelination within the white matter showing anti-inflammatory and antioxidant properties. It also provides anti-excitatory properties through the modulation of gamma-aminobutyric acid (GABA) and glutamate receptors. Additionally, it improves mitochondrial integrity, upregulating antioxidant enzymes. Due its pleiotropic neuroprotective mechanisms, it shows the distinctive ability to limit injury during all phases of hypoxic-ischemic neuronal damage [42,43]. Initially, many animal models of the neonatal brain were used to assess the neuroprotective effects of melatonin [39,41,44]. In 2001, some authors described, for the first time, the decrease in malondialdehyde and nitrite/nitrate levels in the serum of infants with HIE to whom melatonin was administered within the first 6 h of life [45]. Afterward, in 2015, Aly et al. [46] proved that treatment with melatonin and TH in 30 infants with moderate to severe HIE reduced oxidative stress, the number of seizures and white matter lesions, and improved neurodevelopmental outcomes at 6 months of age (*p*-value < 0.001). In that clinical setting, 5 doses of 10 mg/kg/day of melatonin were administered orally, in addition to TH. The small sample of patients was a clear limitation of that study; electroencephalogram (EEG), brain magnetic resonance imaging (MRI), and measuring the serum concentrations of SOD and NO were frequently used to evaluate the participants. At 5 days, the TH + melatonin group showed a decline in NO (*p*-value < 0.001) and SOD (*p*-value = 0.004) values [46]. Oral melatonin has rapid absorption but low bioavailability with great interindividual variability, primarily referred to as high first-pass metabolism in the liver. The intravenous formulation has higher bioavailability [42]. Recently, Balduini et al. [47] demonstrated that the maximum safe and effective dose of melatonin for infants with HIE treated with TH was 5 mg/kg, depending on the delivery method. Furthermore, this study showed that cooling did not affect the pharmacokinetics of melatonin. Carloni et al. [48] studied the pharmacokinetics of the oral administration of melatonin in preterm newborns and demonstrated that a single repeated oral administration every 12/24 h was effective in acquiring and maintaining neuroprotective blood concentrations of melatonin. Animal studies have demonstrated that melatonin has to be administered from 10 min to 2 h after the hypoxic-ischemic injury to show its neuroprotective properties [42]. Therefore, melatonin should be administered as soon as possible. Melatonin is irregularly soluble in water, so is commonly mixed with an excipient (mostly ethanol) to achieve a solution with the correct concentration of melatonin [42]. Since ethanol could have neuroprotective effects and boost GABAergic neurotransmission at a low dosage, its use as a solubility enhancer could have been a bias in former pre-clinical and clinical trials [42]. Therefore, in 2019, Robertson et al. [49] demonstrated the neuroprotective effects of an ethanol-free melatonin formulation at high dosage (15 mg/kg) in combination with cooling in an animal model of HIE. In 2018, Ahmad et al. [50] released the results of a randomized control trial using TH alone or TH plus melatonin on 80 asphyxiated infants. The melatonin plus hypothermia group showed an improved survival rate. Jerez-Calero et al. [51] reported development at 18 months, showing a composite cognitive score that was significantly higher (*p* < 0.05) in the melatonin (5 mg/kg intravenously for three consecutive days) and TH group versus the placebo and TH group. Nevertheless, this difference between the two groups was not present at 6 months of age, and there were no statistical differences for the other components of neurologic development at both 6 and 18 months. Melatonin can easily cross the placenta; hence this ability has led to the assessment of maternal intrapartum administration to reduce the damage of intrauterine fetal asphyxia [41]. A decrease in lipid peroxidation products has only been reported in animal models [52]. Since perinatal asphyxia is often a consequence of an abrupt and random event that could limit the feasibility for research recognition following clinical implementation of this approach [8]. According to clinicaltrials.gov (accessed on 3 July 2024) [53], an USA early phase 1 trial (NCT02621944) is currently recruiting an estimated number of 70 newborns: its main aims are to identify the maximum tolerated dose of melatonin through the oral route, study its pharmacokinetics, and evaluate neurological outcome assessment. As we can see, further studies are warranted. Table 2 shows the results of some relevant clinical trials investigating melatonin-treatment in HIE.

### 4.2. Allopurinol

Allopurinol (4-hydroxy-pyrazole(3,4-d) pyrimidine) is a competitive inhibitor of the enzyme xanthine oxidase, which catalyzes the oxidation of both hypoxanthine and xanthine. It lowers the degradation of purines (especially adenosine), has antioxidant effects, and finally, it reduces brain harm in preclinical and clinical trials of HIE [54,55,56,57]. Moreover, at high concentrations, it acts as an iron-chelator and direct scavenger of free radicals [58,59]. Allopurinol can quickly cross the placenta and the blood–brain barrier and is rapidly metabolized to oxypurinol by xanthine oxidase (XO) and aldehyde oxidoreductase (AOR). Oxypurinol is eliminated unmodified through the kidneys. Allopurinol and oxypurinol have different half-lives: the first 1–2 h, and the second 25–30 h [59]. In 2007, Gunes et al. [60] administered 40 mg/kg of allopurinol (40 mg/kg/day, 3 days, intravenously) within 2 h from birth in 30 asphyxiated term newborns and found improvements in the neurodevelopmental outcomes in the treatment group. In the ALLO-trial (NCT00189007) [61], the administration of intravenous allopurinol (dose of 500 mg) to mothers during the delivery of fetuses with hypoxia or incipient hypoxia (abnormal or non-reassuring fetal heart rate trace or an abnormal fetal blood scalp sampling, pH < 7.20) reduced the cord blood levels of the S-100B protein. The effect was stronger in female neonates, leading to the hypothesis of a potential gender-related effect. Allopurinol was demonstrated to be safe for both neonates and mothers in comparison to the placebo group (who received mannitol). However, the results of the trial were inconclusive for other assessed data [61,62]. In 2011, Kaandorp et al. [63] studied the neurological outcomes of infants with moderate asphyxia (defined by α-EEG) treated with high dose of allopurinol (40 mg/kg intravenously, one dose every 12 h) within 4 h of birth. The treatment with allopurinol was associated with a decreased risk of death or severe long-term disability (at 4–8 years of age) and did not show negative side effects. The two mentioned studies were performed before the routine introduction of TH [61,63].

Nowadays, a blinded randomized placebo-controlled multicentric clinical trial (ALBINO TRIAL, NCT03162653, phase III) [53,64] is currently recruiting an estimated number of 760 newborns. The study will compare allopurinol to a placebo, mannitol. Allopurinol will be administered in two doses: first dose (20 mg/kg), given as soon as intravenous access is established and no later than 30 min postnatally, and the second dose (10 mg/kg) 12 h thereafter. The second dose will only be administered to infants on TH. The aim of this study is to assess death, severe neurodevelopmental impairment, and survival without severe neurodevelopmental impairment at 24 months of age [65]. The study is supposed to end in 2026. Table 3 shows the results of some relevant clinical trials investigating allopurinol treatment in HIE.

### 4.3. Erythropoietin (EPO)

Erythropoietin (EPO) is a glycoprotein hormone that has several erythropoietic and non-erythropoietic roles. Its synthesis is hepatic in fetuses, while in infants, it is synthesized by the kidney and the developing brain, where it acts as a growth factor and neuroprotective agent [56,58]. The production of EPO is regulated through hypoxic cellular responses: typically, it increases in conditions of low oxygen levels [65]. Its activity, through the engagement of EPO receptors present in neurons and glia, as a potent anti-apoptotic agent and as an anti-inflammatory and antioxidant molecule, are the rationale of its use in HIE. Moreover, EPO can promote long-term repair processes such as angiogenesis, oligodendrogenesis, and neurogenesis [58,66]. In preclinical studies, the combined administration of EPO and TH has provided inconsistent results. In clinical studies, in newborns with HIE, the safe dose of EPO ranged from 300 to 2500 IU/kg. Low doses of EPO may be effective in infants with moderate damage and seem to be correlated with a decreased threat of disability or death [65]. In 2009, an Egyptian trial (NCT00945789) showed that higher doses (2500 IU/kg/die subcutaneously for 5 days) administered within the first 24 h of life reduced the number of seizures and neurologic abnormalities at 6 months of life [67]. In 2016, a U.S. phase II double-blinded placebo-controlled trial (Neonatal Erythropoietin and Therapeutic Hypothermia Outcomes in Newborn Brain Injury (NEATO); NCT01913340) showed that EPO + TH-treated infants (1000 UI/kg intravenously at specific intervals in the first week of age with the first dose within 24 h) [68] had inferior incidence of moderate or severe brain, subcortical, and cerebellar harm than the placebo + TH group in the first week of life in brain MRI scans [53]. Moreover, the around 1 year of life EPO-treated infants had a better motor performance than the placebo-treated ones. This was also assessed as the key importance of placental pathology in the EPO treatment response in HIE [68]. The Erythropoietin for Hypoxic Ischemic Encephalopathy in Newborns trial (PAEAN; NCT03079167) [53] and High-Dose Erythropoietin for Asphyxia and Encephalopathy trial (HEAL; NCT02811263) [53] have shown that combined therapy with EPO and TH in infants with HIE did not lower the risk of death nor improve the neurological outcomes compared to the placebo; however, in the combination therapy EPO + TH groups, there were higher rates of serious adverse events [65]. Another evaluation of the data of HEAL stated that EPO did not lower the biomarkers of neuroinflammation or brain injury in asphyxiated infants; nevertheless, the estimation of 2-year outcome was, within reasonable limits, improved by the assessment of circulating biomarkers (C5a, IL-6, and neuron-specific enolase, NSE, at the baseline; IL-8, tau and ubiquitin carboxy-terminal hydrolase-L1 at day 4) [69]. Darbepoetin is an EPO analog, that is safe and effective as an EPO, but has higher stability and biological activity, a longer half-life, and a decreased receptor affinity [65]. A recent meta-analysis [70] showed that using EPO, both recombinant and not, would not enhance the risk of side effects (such as thrombocytopenia, hypotension, hepatic adrenal injury); however, in neonates with HIE treated with EPO, there was not a significant reduction in death and neurological impairment. Table 4 shows the results of some relevant clinical trials investigating EPO-treatment in HIE.

### 4.4. N-Acetylcysteine

N-Acetylcysteine (NAC) is a membrane-permeable precursor of cysteine that regulates oxidative stress by scavenging free radicals and maintaining glutathione levels [58,62]. NAC crosses the placenta and does not have teratogenic effects [58]. Treatment with this drug has been studied antenatally and postnatally in infants exposed to chorioamnionitis [71]. NAC has shown efficacy in providing effective neuroprotection in animal models of severe HIE [72,73]. Calcitriol is a neuro-steroid implicated in myelination, neuroplasticity, and neurological development. It also induces the synthesis of glutathione reductase, and therefore increases reduced glutathione [72]. A recent clinical trial (NCT 04643821) [53] investigated the intravenous administration of NAC (25–40 mg/kg/dose; twice per day) and vitamin D (0.03–0.05 mg/kg/dose; once or twice per day according to the group) for ten days in a small cohort of infants with moderate or severe HIE in hypothermia and normothermia. This therapy was initiated within 4–9 h after birth. The treated infants showed no major side effects; hypercalcemia due to vitamin D was reported, but it was manageable by stopping the administration of active vitamin D and/or reducing calcium supplementation in parenteral nutrition. This treatment resulted in the rapid synthesis of reduced glutathione and an increase in energetics in the central nervous system. The study participants were evaluated through MRI, magnetic resonance spectroscopy, and reduced glutathione levels. In the 24 treated asphyxiated infants, there was no evidence of cerebral palsy, autism, or neurocognitive impairment at 1–2 years of age [72,74]. Although more numerous trials are needed, NAC seems promising in the near future for neonatal HIE.

### 4.5. NOS Inhibitors

NO is produced by different NOS isoforms: neuronal (nNOS), endothelial (eNOS), and inducible (iNOS). eNOS has a neuroprotective effect that promotes perfusion of the brain if demanded [75]. Hypoxic conditions upregulate those enzymes, expressed in neurons, astrocytes, and endothelial cells; hence NOS inhibition has been studied as a therapeutic strategy for HIE [76]. Some molecules of this class are aminoguanidine, 7-nitroindazole, NG-nitro-L-arginine, and 2-iminobiotin (2-IB) [8]. 2-IB is a vitamin H or B7 analog and incorporates guanidine and free carboxyl groups that allow 2-IB to bind within the active site of NOS. In preclinical studies, 2-IB has been shown to reduce the production of NO, inhibiting iNOS and nNOS activity, in a concentration-dependent way [66]. However, the action of 2-IB seems to result from the blockage of the cytochrome C/caspase-3 apoptotic cascade rather than from NOS inhibition [77]. In the last decade, two small phase 2 clinical studies (NCT01626924; EudraCT 2014-004265-25) [53,78] have investigated the safety and pharmacokinetics of 2-IB in near term newborns with moderate or severe HIE. 2-IB was administered intravenously, and the 2-IB dose was set as 0.08–0.16 mg/kg every 4 or 6 h for 24 or 48 h without any severe side effects [78]. Further studies are needed.

### 4.6. Magnesium (Mg) Sulfate

Magnesium sulfate is an inorganic salt that is used in several clinical conditions [56]. It has anti-excitotoxic and anti-oxidative properties, blocking the NMDA receptor and downregulating proinflammatory cytokines [66]. El Farargy et al. [79] observed that the serum S100B concentration was notably lower in the magnesium and melatonin group (30 infants with moderate HIE, Sarnat II) compared to the melatonin only group (30 infants with moderate HIE, Sarnat II), indicating a synergistic effect of magnesium and melatonin in neuroprotection. At high doses, magnesium sulfate has a significant risk of hypotension [75]. In 2020, a Pakistani phase 2 study (NCT04705142) [53] enrolled 200 newborns with HIE in a low-income country setting. Magnesium sulfate or the conventional treatment (supportive care) was administered intravenously at the dosage of 250 mg/kg at 6, 24, and 48 h of age. The aim of the study was to demonstrate the reduction in immediate complications as well as a reduction in mortality and hospital stay in participants at two weeks of age. The neurological assessment at discharge (evaluated through the length of seizures, the ability to suck feed, and the presence of neurological condition) was significantly better in the magnesium sulfate group than in the control [80]. According to clinicaltrials.gov [53], a Pakistani interventional phase IV study in which the participants will receive either magnesium sulfate or a placebo is currently in progress (NCT06342362). The study population will be all term and near-term newborns with moderate to severe HIE reaching the neonatal intensive care unit (NICU) within 6 h of life. The aim of the study is to measure the neuroprotective effects at 6 months of age.

### 4.7. Sovateltide

Sovateltide is a synthetic analog of endotelin-1 and is a highly specific endothelin B receptor agonist. It repairs and regenerates neuronal cells after an injury, with a consequent improvement in neurological and motor functions [81]. It has been shown to be safe, well-tolerated, and effective in clinical trials in adults suffering from acute cerebral ischemic stroke [53]. Since HIE and ischemic stroke have a common pathophysiology, this drug could also be efficacious in HIE. Moreover, this molecule promotes angiogenesis and neurogenesis, enhancing neuronal plasticity and decreasing neuronal cell loss secondary to HIE [65]. An Indian multicenter, randomized, double-blind, placebo-control phase-II trial (NCT05514340) is currently in progress: TH+0.3 µg/kg of sovateltide will be compared to TH+the same dose of placebo [65]. Further studies are warranted.

### 4.8. Stem Cells

Stem cell treatment (mesenchymal and neural stem cells, mononuclear cells, oligodendrocyte progenitor cells, hematopoietic and inducible pluripotent stem cells, endothelial cells) has shown promising results in animal models [82], therefore, several studies in newborns have been carried out [57,65]. Their mechanism of action is due to their ability to regulate immune response through their interaction with effector immune cells distant from the brain (mostly in the spleen) and increase neurogenesis and cellular proliferation in the interaction with injured brain tissue. Therefore, stem cell treatment in HIE could help in the reparation and regeneration of damaged brain tissue [58]. A phase I U.S. study (NCT00593242), completed in 2017, achieved promising results with the autologous transplantation of umbilical cord blood cells: the stem cell + TH group showed better neurological development than the TH alone group [83]. The NEOSTEM trial (Neonatal Hypoxic Ischemic Encephalopathy: Safety and Feasibility Study of a Curative Treatment with Autologous Cord Blood Stem Cells) will enroll 20 infants that will be treated with 5 × 10^7^/kg autologous mononuclear cells from umbilical cord blood, and its aims will be to assess the side effects due to stem cell preparation until 2 years of age and to assess neurodevelopmental function until 2 years of age [53]. Finally, NCT06427642, a clinical trial currently being registered [53], will enroll 120 infants with HIE, bronchopulmonary dysplasia, or short bowel syndrome who will be treated with either mononuclear cells obtained from umbilical cord blood+TH or TH only. The aims of the study will be to evaluate the incidence of adverse reactions, the incidence of complications, the frequency of seizures via EEG, ventilator support time, and oxygen demand.

Table 5 summarizes the results of the main clinical studies carried out on the molecules previously described.

**Figure 2:**
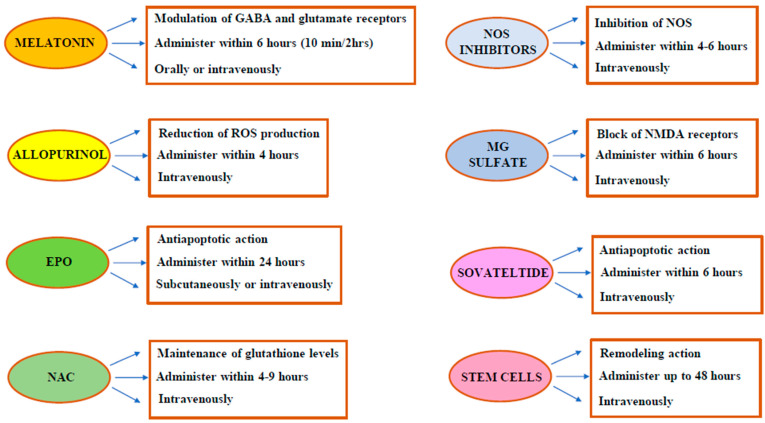
A schematization of the mechanism of action and dosage of the main antioxidants that could be used during HIE. GABA: gamma-aminobutyric acid; ROS: reactive oxygen species; NOS: nitric oxide synthase; NMDA: N-methyl D aspartate.

Figure 2 shows a schematization of the mechanism of action and dosage of the main antioxidants that could be used during HIE.

### 4.9. Other Promising Antioxidants: Miscellany

Several antioxidants were investigated for neonatal HIE and showed interesting potential effects in animal models.

1. Docosahexaenoic Acid (DHA)

DHA is a polyunsaturated fatty acid enriched in phospholipids [75]. It has free radical scavenging ability and decreases glutamate excitotoxicity, reduces NO, and increases antioxidant enzymatic activities. The molecule showed neuroprotective effects in animal models [8].

The intake of DHA during pregnancy (especially in the third trimester) is very important due to its beneficial effects on the development of the central nervous system (CNS) and the retina of the fetus [84,85].

2. Lactoferrin

Lactoferrin is a fundamental protein in colostrum and human milk; it plays an important role in postnatal development. Its iron chelating, anti-inflammatory, antioxidant, and antiapoptotic properties have been stated in numerous models of perinatal and neonatal hypoxia and ischemia [86].

3. Lutein

Lutein is a member of the family of carotenoids and has a documented antioxidant activity [75].

4. Vitamins C and E

These vitamins are essential nutrients and are considered to be the most important antioxidants obtained through diet [75]. Vitamin C (ascorbic acid) has shown a neuroprotective effect after hypoxic-ischemic injury in immature rats [87]. Although a clinical study in newborns with HIE in the early 2010s seemed promising, other clinical trials have not confirmed those results [88]. Vitamin E (α-tocopherol) has numerous antioxidative properties [89]. A preclinical study in a gyrencephalic ferret brain slice model of neonatal hypoxia-ischemia has shown that vitamin E provides neuroprotection [90].

5. Deferoxamine

Deferoxamine is an iron chelator produced by *Streptomyces pilosus*, which binds free iron and boosts its elimination. Deferoxamine can cross the blood–brain barrier. Studies in animal models of HIE (rats, lambs, piglets) have documented its ability to reduce brain injury and improve cerebral metabolism [23,56,75].

6. Dihydroartemisinin

Dihydroartemisinin, derived from the well-known antimalaric artemisinin, has shown antioxidant and anti-inflammatory activities in animal models [91].

7. Edaravone

Edaravone (3-methyl-1-phenyl-pyrazolin-5-one) scavenges free radicals and has shown several antioxidant properties in animal models of HIE and in a small sample of pediatric patients with cerebral infarction (not HIE-correlated), with promising neuroprotective effects and without significant adverse ones [8,92].

8. Exendin-4

Exendin-4 is a glucagon-like peptide 1 receptor agonist. In vitro and in vivo data have documented its anti-inflammatory, anti-apoptotic, antioxidant, and neurotrophic effects. One preclinical study conducted in neonatal mice showed promising results [12,93].

9. Carnosine

Carnosine is an endogenous dipeptide (β-alanyl-l-histidine) and is normally found in both cardiac and skeletal muscles and in the nervous tissue. Recently, laboratory and animal studies have documented its neuroprotective effects against HIE [94].

Several natural plant products have shown promising effects, among them we recall resveratrol, cannabidiol, and caffeine [12,56,57,95].

Others interesting potential future antioxidant therapies are progesterone [96], molecular hydrogen [97], hyperbaric oxygen therapy [23], and mitochondrial therapy [23].

Table 6 shows animal models of potential antioxidant use in HIE.

## 5. Conclusions and Future Perspectives

HIE has a massive impact worldwide for its incidence and for the important neurological and non-neurological sequelae. In high-income countries, TH has become the standard of care for asphyxiated newborns, who are eligible for this treatment (>35 wks, >1800 gr, and within 6 h of life, in the presence of asphyxia, demonstrated by pH and/or base excess values, neurological examination abnormalities, and aEEG/EEG anomalies) [34,35,36,37,38]. Nevertheless, TH has not completely solved HIE, since neonates with moderate or severe HIE treated with TH still experience devastating complications such as combined death or moderate/severe disability. There is also a concern that TH may not be effective in low- and middle-income countries. Therefore, it is essential to study new pharmacological treatment options in conjunction with TH in high-income countries or alone in countries where TH is not feasible. Many molecules have been studied. Melatonin, allopurinol, N-Acetylcysteine, and Mg sulfate, among others, show promise, although more studies and long-term follow-ups are needed to determine the correct dosage and timing and to add them in daily clinical practice. Melatonin and Mg sulfate stand out as a highly relevant options as readily accessible treatments in low- and middle-income countries. Moreover, in high-income countries, antioxidant therapies could play a role in infants that have suffered hypoxic-ischemic neuronal damage, but do not fit the criteria for TH. Although HIE remains a global challenge for the next few years, new antioxidant drugs give hope for a better future for asphyxiated neonates and their families.

## Figures and Tables

**Table 1:** Summary of recommendations to perform TH ([34,35] modified). GA: gestational age; BW: birth weight; SUPC: sudden unexpected postnatal collapse; aEEG: amplitude integrated electroencephalography.

Baseline Characteristics	Asphyxia (Criterion A)	Neurological Examination Abnormalities (Criterion B)	aEEG/EEG Anomalies (Criterion C)	Recommendation Level
GE > 35 wks BW > 1800 g Timing <6 h	Strong	Strong	Strong	Strong
GE > 35 wks BW > 1800 g Timing < 6 h	Strong	Strong	Not Evaluable/Not Available	Strong
GE = 35 wks BW > 1800 g Timing < 6 h	Strong	Strong	Strong	Weak
GE >35 wks BW > 1800 g Timing < 6 h	Not Satisfied	Strong	Strong	Research Context
GE > 35 wks BW > 1800 g Timing 6–24 h	Strong	Strong	Not Applicable	Research Context
SUPC GE >35 wks BW > 1800 g Timing < 6 h	Strong	Strong	Strong	Research Context

**Table 2:** Results of some relevant clinical trials investigating melatonin treatment in HIE. TH: therapeutic hypothermia; HIE: hypoxic ischemic encephalopathy; SOD: superoxide dismutase; NO: nitric oxide.

Melatonin					
Authors	Publication Date	No. of Patients	Dosages/Formulations Used	PMID	Conclusions
Fulia et al. [45]	2001	20 newborns with perinatal asphyxia were investigated	10 asphyxiated infants received 8 doses of 10 mg each separated by 2-h intervals of melatonin orally; 10 asphyxiated infants received placebo.	11703564	Reduction in malondialdehyde and nitrite/nitrate levels in asphyxiated children treated with melatonin compared to the placebo.
Aly et al. [46]	2015	30 neonates affected by HIE were investigated	15 asphyxiated infants received both TH and melatonin in a dose of 10 mg/kg daily for a total of five doses orally; 15 asphyxiated infants received only TH.	25393080	Combination of melatonin and TH in moderate to severe HIE, reduced oxidative stress (SOD and NO), the number of seizures and white matter lesions, improving neurodevelopmental outcomes at 6-months of age.
Ahmad et al. [50]	2018	80 newborns with perinatal asphyxia were investigated	40 asphyxiated infants received both TH and melatonin in a dose of 10 mg in single-shot; 40 asphyxiated infants received only TH.	30108392	Combination of melatonin and TH improved survival rate.
Jerez-Calero et al. [51]	2020	25 newborns with perinatal asphyxia were investigated	12 asphyxiated infants received both TH and melatonin in a dose of 5 mg/kg for 3 days intravenously; 13 asphyxiated infants received only TH.	32168305	Combination of melatonin and TH improved the composite cognitive score (*p*-value < 0.05) at 18-months of age.

**Table 3:** Results of some relevant clinical trials investigating allopurinol treatment in HIE.

Allopurinol					
Authors	Publication Date	No. of Patients	Dosages/Formulations Used	PMID	Conclusions
Gunes et al. [60]	2007	30 newborns with perinatal asphyxia were investigated	30 asphyxiated infants received 3 doses of 40 mg/kg/day of allopurinol intravenously; first dose within 2 h of birth.	17162192	Improvement in neurodevelopmental outcomes.
Torrance et al. [61] ALLO-trial NCT00189007	2009	53 mothers during the delivery of 54 fetuses with hypoxia or incipient hypoxia	26 mothers received 500 mg of allopurinol intravenously; 27 mothers received placebo (mannitol).	19564319	Reduction in S-100β protein level in cord blood of treated pregnant women. Allopurinol was safe for both the neonates and the mothers.
Kaandrop et al. [63]	2012	22 children with history of perinatal asphyxia were investigated	13 children were treated with high doses of allopurinol (40 mg/kg twice per day) within 4 h of birth. 9 children received the placebo.	22102633	Decreased risk of death or severe long-term disability at 4–8 years of age without significant side effects.

**Table 4:** Results of some relevant clinical trials investigating EPO treatment in HIE. MRI: magnetic resonance imaging; TH: therapeutic hypothermia; HIE: hypoxic ischemic encephalopathy.

Erythropoietin					
Authors/ Study	Publication Date	No. of Patients	Dosages/Formulations Used	PMID	Conclusions
Elmahdy et al. [67] NCT00945789	2010	30 infants with HIE; 15 healthy infants	15 infants with HIE received 2500 IU/kg/die of EPO, subcutaneously for 5 days, within the first 24 h of life. 15 infants with HIE received the placebo.	20385632	Improvement in electoencephalographic backgrounds and decreased NO concentrations at two weeks of age in the treated group. Reduction in the incidence of seizures and neurological abnormalities at 6 months of age in the treated group.
Wu et al. [68]	2016	50 infants with moderate/severe encephalopathy	24 infants received 1000 IU/kg of EPO at 1, 2, 3, 5 and 7 days of age plus TH. 26 infants received placebo at 1, 2, 3, 5, and 7 days of age plus TH.	27244862	Brain MRI scans of the EPO+TH group showed lower incidence of moderate/severe brain injury, subcortical injury and cerebellar injury than the placebo+TH group in the first week of life; around 1 year of life EPO-treated infants demonstrated better motor performance than the placebo-treated infants.
PAEN, NCT03079167 [53]	2024	300 infants with HIE	150 infants received 1000 IU/kg of EPO at 1, 2, 3, 5, and 7 days of life plus TH; 150 infants received the placebo at 1, 2, 3, 5 and 7 days of life plus TH.	/	EPO administration to newborns undergoing TH for HIE did not result in a lower risk of death or neurodevelopmental impairment than the placebo and was associated with a higher rate of serious adverse events.
Pan et al. [70]	2023	1262 infants with HIE in 11 different studies	636 infants treated with different dosages of EPO that ranged from 200 IU/kg to 1000 IU/kg (subcutaneously or intravenously); 626 infants treated with the placebo.	36699298	The metanalysis showed that using EPO would not increase the risk of adverse events, however, it is not beneficial for reducing death and improving neurological impairment in HIE-affected neonates.

**Table 5:** Results of the main relevant clinical trials investigating NAC, NOS-inhibitors, Mg sulfate, sovateltide, and stem cell treatment in HIE.

N-Acetylcysteine					
Authors	Publication Date	No. of Patients	Dosages/Formulations Used	PMID	Conclusions
Jenkins et al. [72]	2021	30 infants with moderate or severe HIE in hypothermia	10 newborns received 25 mg/kg dose of NAC twice per day intravenously + 0.05 ug/kg/dose of calcitriol D twice per day, for 10 days; 10 newborns received 25 mg/kg dose of NAC twice per day intravenously + 0.03 ug/kg/dose of calcitriol D twice per day for 10 days; 10 newborns received 40 mg/kg dose of NAC twice per day intravenously + 0.03 ug/kg/dose of calcitriol D once a day for 10 days.	34572976	The study participants were evaluated through MRI, magnetic resonance spectroscopy, and reduced glutathione levels. In all 30 treated asphyxiated infants, there was no evidence of cerebral palsy, autism, or neurocognitive impairment at 1–2 years of age.
Moss et al. [74]	2018	24 infants with moderate or severe HIE in hypothermia	Newborns received daily intravenous NAC and calcitriol infusions as NAC 25–40 mg/kg every 12 h and calcitriol 0.03–0.1 mg/kg/day from 6 h of life to 10 days or discharge.	29561203	In all 24 treated asphyxiated infants, the neuroprotective effect of NAC and calcitriol was demonstrated by evaluating the brain MRI.
**NOS Inhibitors**					
NCT01626924 [78]	2016	6 near-term newborns with moderate or severe HIE	0.08–0.16 mg/kg of 2-IB have been administered every 4 or 6 h intravenously, for 24 or 48 h.	/	2-IB was administered without any severe side effects.
**Magnesium (Mg) Sulfate**					
El Farargy et al. [79]	2019	60 infants with moderate HIE (Sarnat II) have been recruited	30 infants received 25 mg/kg/day of MgSO_4_ intravenously, at days 0 and 1; then, they received 10 mg/kg/day of melatonin for 5 days, orally. 30 infants received only 10 mg/kg/day of melatonin for 5 days, orally.	31609707	Serum S100B concentration, which correlates with the severity of HIE, was significantly reduced in the infants who received both magnesium and melatonin, suggesting a synergistic effect of magnesium and melatonin in neuroprotection.
**Sovateltide**					
NCT05514340 [65]	Ongoing trial	40 infants with HIE	20 infants treated with TH+0.3 µg/kg/dose of sovateltide, intravenously, every 3 h on days 1, 3 and 6; 20 infants treated with TH+ the same dose and scheme of placebo.	/	The primary aim of the study is to evaluate the percentage of patients with death or disability (moderate or severe) in the sovateltide group compared to the control group.
**Stem Cells**					
Cotten et al. [83]	2014	52 infants with HIE	52 infants received 5 × 10^7^ cells/kg of autologous cord blood for 4 infusions plus TH.	24388332	74% of the newborns that received stem cells survived with scores of 85 or higher in the Bayley scales, compared to 41% of the newborns treated with TH alone (historical controls).
NEOSTEM trial NCT02881970 [clinicaltrials.gov]	Ongoing trial	20 infants	All children will receive 5 × 10^7^/kg of autologous mononuclear cells from umbilical cord blood.	/	The aims will be to evaluate adverse clinical or paraclinical event rates due to stem cell preparation until 2 years of age and to assess neurodevelopmental function until 2 years of age
NCT06427642 [clinicaltrials.gov]	Ongoing trial	120 infants with HIE, bronchopulmonary dysplasia, or short bowel syndrome	Therapy with mononuclear cells obtained from umbilical cord blood+TH will be compared with TH only.	/	The aims of the study will be to evaluate the incidence of adverse reactions, the incidence of complications, the frequency of seizures via EEG, ventilator support time, and oxygen demand.

**Table 6:** Potential antioxidant use in HIE (animal models).

Potential Antioxidant Use in HIE (Animal Models)	
Dietary elements	Docosahexaenoic acid [8], lactoferrin [86], lutein [75], vitamin C [87], vitamin E [90]
Drugs	Deferoxamine [75], dihydroartemisinin [91], edaravone [92], exendin-4 [93], mitochondrial therapy [23]
Endogenous substances	Carnosine [94], progesterone [96]
Natural plant products	Caffeine, cannabidiol, resveratrol [56,57,95]
Others	Hyperbaric oxygen therapy [23], molecular hydrogen [97]

## Data Availability

No new data were created or analyzed in this study.
